# *Constitutive Expressor of Pathogenesis-Related Genes5 *affects cell wall biogenesis and trichome development

**DOI:** 10.1186/1471-2229-8-58

**Published:** 2008-05-16

**Authors:** Ginger Brininstool, Remmy Kasili, L Alice Simmons, Viktor Kirik, Martin Hülskamp, John C Larkin

**Affiliations:** 1 Louisiana State University, Department of Biological Sciences, Baton Rouge, LA, USA; 2 University of Köln, Botanical Institute III, Köln, Germany; 3 Department of Plant Biology, Carnegie Institution of Washington, Stanford, CA, USA

## Abstract

**Background:**

The Arabidopsis thaliana *CONSTITUTIVE EXPRESSOR OF PATHOGENESIS-RELATED GENES5 *(*CPR5*) gene has been previously implicated in disease resistance, cell proliferation, cell death, and sugar sensing, and encodes a putative membrane protein of unknown biochemical function. Trichome development is also affected in *cpr5 *plants, which have leaf trichomes that are reduced in size and branch number.

**Results:**

In the work presented here, the role of *CPR5 *in trichome development was examined. Trichomes on *cpr5 *mutants had reduced birefringence, suggesting a difference in cell wall structure between *cpr5 *and wild-type trichomes. Consistent with this, leaf cell walls of *cpr5 *plants contained significantly less paracrystalline cellulose and had an altered wall carbohydrate composition. We also found that the effects of *cpr5 *on trichome size and endoreplication of trichome nuclear DNA were epistatic to the effects of mutations in *triptychon *(*try*) or overexpression of *GLABRA3*, indicating that these trichome developmental regulators are dependant on *CPR5 *function for their effects on trichome expansion and endoreplication.

**Conclusion:**

Our results suggest that *CPR5 *is unlikely to be a specific regulator of pathogen response pathways or senescence, but rather functions either in cell wall biogenesis or in multiple cell signaling or transcription response pathways.

## Background

Mutations in the *CONSTITUTIVE EXPRESSOR OF PATHOGENESIS-RELATED GENES5 *(*CPR5*) gene of *Arabidopsis thaliana *are highly pleiotropic, affecting pathogen responses, cell proliferation, cell expansion, and senescence. The gene was initially identified based on the constitutive pathogen response phenotype of the mutants [1,2], and appears to act just downstream of pathogen recognition and upstream of salicylic acid in *NPR1*-dependent disease resistance [1]. In addition, Boch and co-workers [2] showed that *CPR5 *activates pathogenesis-related (PR) gene expression in the *RPS2*-mediated pathway and not the *RPM1*-mediated pathway. However, *CPR5 *appears to play a broader role in plant growth and development as well, because *cpr5 *mutants exhibit defects in cell proliferation and cell expansion, and the gene has been hypothesized to play a role in programmed cell death [3]. In addition, *cpr5 *mutants are hyper-responsive to glucose and sucrose and prematurely accumulate senescence-regulated transcripts [4]. The *CPR5 *gene encodes a putative membrane protein with five putative transmembrane domains at the carboxy-terminus, a putative bipartite nuclear localization signal at the amino-terminus, and no sequence similarity to other known proteins [3,4].

In contrast to other constitutive pathogen response mutants, *cpr5 *mutations affect trichome morphology. The trichomes on Arabidopsis leaves are specialized single cells that project from the epidermis, and in wild-type they have an unusual branched shape. In addition, wild-type trichomes replicate their DNA without division during development in a process called endoreplication or endoreduplication, reaching nuclear DNA levels of 16C-32C [5,6]. Trichomes of *cpr5 *mutants are smaller and less branched than those of wild-type, and have a lower nuclear DNA content [3]. This trichome phenotype suggests that, unlike other constitutive pathogen response mutants, *CPR5 *may play a more specific role in trichome development.

Arabidopsis trichomes are used as a model of plant cell differentiation and cell biology [7,8], and the control of early trichome development is well-understood. Initiation of trichome development requires a transcription factor complex consisting of the basic-helix-loop-helix transcription factor GLABRA3 (GL3), the Myb transcription factor GLABRA1 (GL1), and the WD-repeat protein TRANSPARENT TESTA GLABRA (TTG). Mutations in these genes result either in the absence of trichomes, or in reduced numbers of trichomes, and interactions among these proteins have been demonstrated in yeast. The TRIPTYCHON (TRY) protein acts as a negative regulator of trichome development, and is thought to prevent neighboring cells from developing as trichomes by diffusing to neighboring cells via plasmodesmata and inhibiting trichome development in a classic lateral inhibition mechanism. TRY has a Myb DNA-binding domain, but lacks a transcription activation domain, and can bind to GL3 in yeast, suggesting that it directly inhibits function of the GL1/GL3/TTG complex in cells neighboring a developing trichome [9].

Several mutants affect endoreplication levels in trichomes, and these mutants reveal that nuclear DNA content, trichome size, and trichome branching are highly correlated, with mutants resulting in higher DNA contents generally having trichomes that are larger and more branched [5,10,11]. Among the genes that control the degree of trichome expansion and endoreplication are the trichome cell fate regulators themselves. Endoreplication is reduced in *gl3 *loss-of-function mutants, and these mutants have smaller trichomes with reduced branching, while *try *mutants have increased levels of trichome endoreplication, and increase trichome size and branching [5]. Trichomes of plants containing the gain-of-function *gl3-sst *allele also have large, extra-branched trichomes with enlarged nuclei indicative of an increased DNA content [9]. These observations indicate that GL3 is required for continued trichome development beyond initiation, and that TRY acts within developing trichomes to limit the extent of expansion and endoreplication, in addition to its role in preventing neighboring cells from developing as trichomes.

To gain insight into the function of CPR5, we examined the role of this gene in the context of the well-understood pathway for trichome development. Here, we show that *cpr5 *mutants have altered cell walls with a reduced cellulose content in leaves as well as in trichomes, a previously unrecognized aspect of the phenotype. We also find that *cpr5 *mutations are epistatic to the extra expansion of trichome cells conditioned by either *GL3 *gain-of function and *try *loss-of-function. The *cpr5 *mutations also increase the number of adjacent trichomes formed due to failure of lateral inhibition signaling in *try *mutants. Our work indicates that the pleiotropy of *cpr5 *mutants is due to a primary role for the gene product in a general cellular process such as cell wall biogenesis or integrity that impinges on many cellular pathways, rather than a specific role in pathogen response signaling or senescence.

## Results

### Mutant phenotype

**Figure 1:**
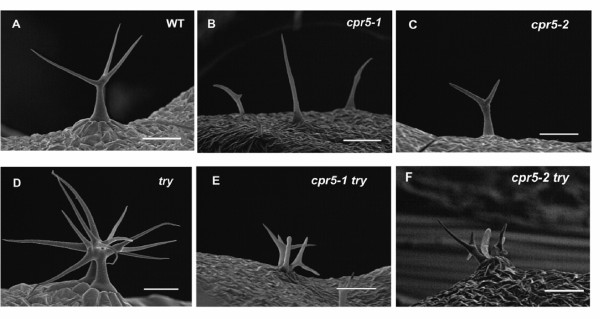
**Trichome phenotypes of *cpr5 *alleles and double mutants with *try*.** Images are scanning electron micrographs; all scale bars are 200 μm. (A) Col-0 wild-type, (B) *cpr5-1*, (C) *cpr5-2*, (D) *try-JC*, (E) *cpr5-1 try-JC *double mutant, (F) *cpr5-2 try-JC *double mutant.

Two recessive alleles were used in this study, *cpr5-1 *[1] and *cpr5-2 *[2]. As described previously by others, the overall size of *cpr5 *mutant plants was smaller than wild-type, cotyledons of *cpr5 *plants senesce sooner than those of wild-type, lesions are present on *cpr5 *rosette leaves, and both leaf epidermal cell size and cell number were greatly reduced in comparison with wild-type [1-4]. For all aspects of the phenotype, *cpr5-1 *plants have a more severe mutant phenotype than *cpr5-2 *mutant plants. The *cpr5-1 *mutation is a missense mutation in the fourth exon (G420D), and the *cpr5-2 *mutation creates a premature stop in the fourth exon at codon 477. Of greatest relevance to this work, trichome branching and size were reduced in plants homozygous for either *cpr5 *allele (Figure 1A, B, C; Table 1). For *cpr5-1 *homozygotes, more than 60% of the trichomes were unbranched, essentially the same fraction of unbranched trichomes that was reported by Kirik et al. [3] for the strong *cpr5-T1 *allele, indicating that *cpr5-1 *results in a loss of function comparable to that of the strongest characterized alleles.

**Table 1:** Effect of *cpr5 *alleles on trichome branching.

	Trichome Branch Points			
Genotype	0	1	2	3
Col	0	3.8	77.3	18.9
cpr5-1	60.9	36.8	2.3	0
cpr5-2	1.1	65.5	33.3	0

For each genotype, branches on a minimum of 400 trichomes were counted.

### *cpr5 *mutants have an altered cell wall

Trichomes of *cpr5 *mutants were more transparent than those of wild-type, and appeared glassy, suggesting that the trichome cell wall of the mutants differed from wild-type trichome cell walls. One readily observable property of plant cell walls is the birefringence they exhibit in polarized light due to the presence of paracrystalline cellulose, a major component of plant cell walls. Paracrystalline cellulose contributes to the high degree of birefringence observed in wild-type trichome cell walls [12]. This birefringence depends on the orientation of the sample relative to the plane of polarization of the illuminating light.

**Figure 2:**
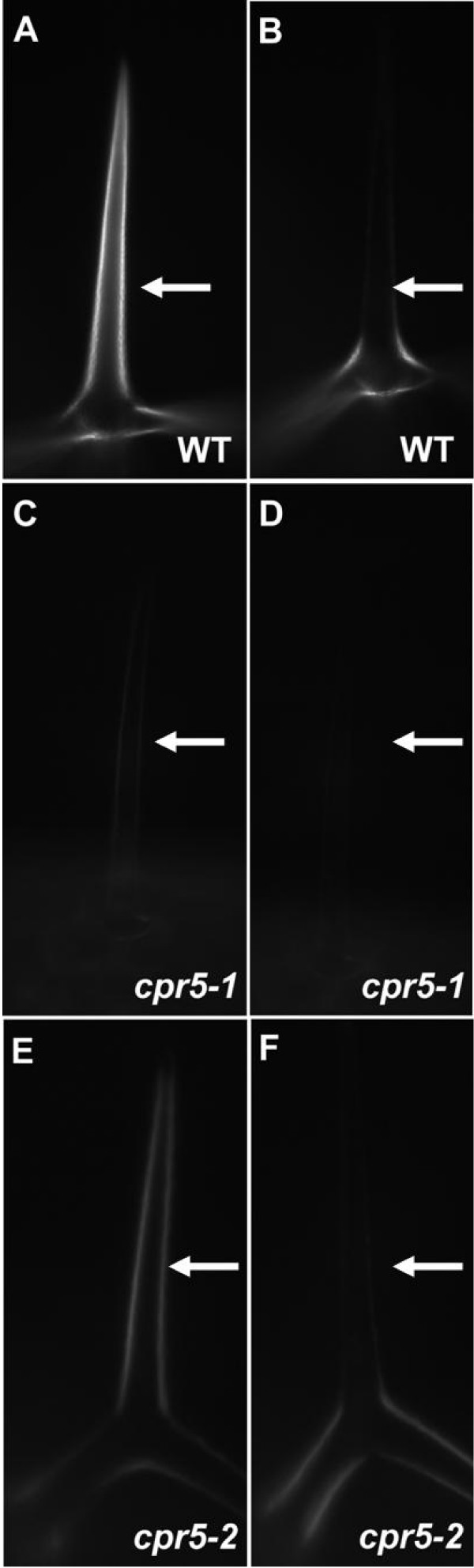
**Reduced birefringence of *cpr5 *mutant trichomes when viewed by polarized light.** Samples were illuminated by plane polarized light and viewed through an analyzer filter oriented at right angles to the polarizing filter. When oriented appropriately relative to the filters, birefringent materials result in the transmission of light through the analyzer. In panels (A), (C), and (E), the trichome branch indicated by the arrow is oriented to transmit maximum light, indicative of the degree of birefringence. In panels (B), (D), and (F), the stage has been rotated relative to the polarizing filters such that the same trichome branch transmits a minimum amount of light. The samples are: Col-0 wild-type, (A) and (B); *cpr5-1*, (C) and (D);*cpr5-2*, (E) and (F).

We examined wild-type and *cpr5 *trichomes by polarized light microscopy. As expected, wild-type trichomes were highly birefringent, indicated by transmission of light when a trichome branch was oriented appropriately relative to the plane of polarization (Figure 2A), whereas *cpr5-1 *trichomes showed little detectable birefringence (Figure 2C), and *cpr5-2 *trichomes exhibited reduced birefringence (Figure 2E). Quantitative comparison between the maximum amount of transmitted light (Figure 2A, C, E) and minimum amount of transmitted light (Figure 2B, D, F) as the specimen was rotated revealed a 36.0 ± 7.8-fold difference for wild-type trichomes, a 2.0 ± 2.0-fold difference for *cpr5-1 *trichomes, and a 16.0 ± 11.0-fold difference for *cpr5-2 *trichomes.

**Figure 3:**
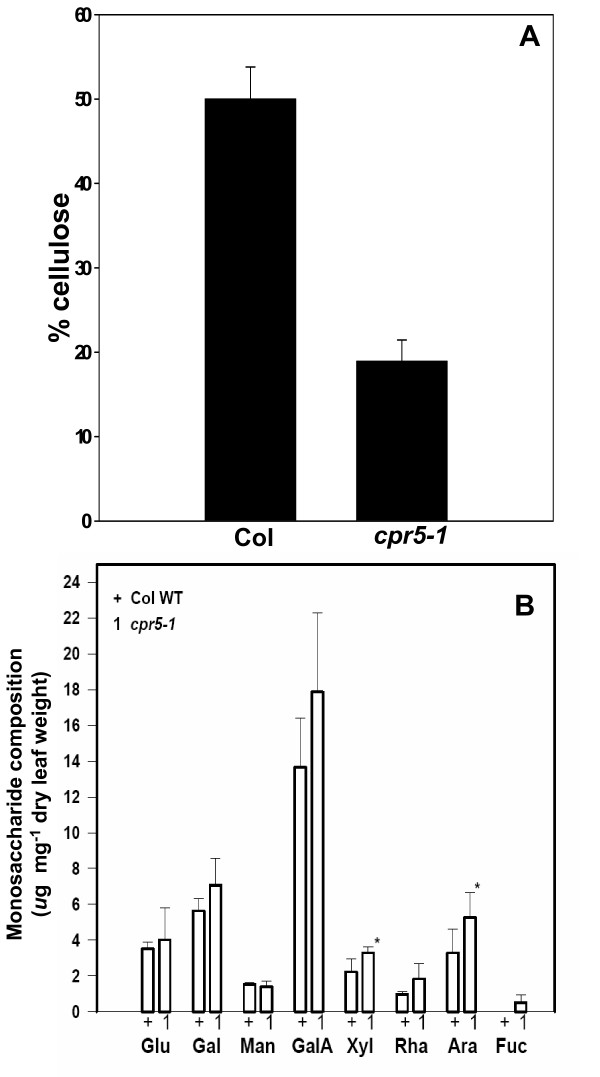
**Trichomes of *cpr5 *plants have an altered cell wall composition.** (A) Paracrystaline cellulose composition of ethanol-insoluble cell walls of rosette leaves, as determined by the Updegraff method [13], N = 3; error bars show standard deviation. * indicates a significant difference from wild-type in a paired t-test (*cpr5-1 *vs. Col-0, p < 0.002). (B) Non-cellulosic monosaccharide composition of rosette leaf cell walls. Data are the mean of three determinations; error bars show standard deviation; * indicates a significant difference in a paired t-test, p < 0.05. Glu = glucose, Gal = galactose, Man = mannose, GalA = galacturonic acid, Xyl = xylose, Rha = rhamnose, Ara = arabionose, Fuc = fucose.

**Figure 4:**
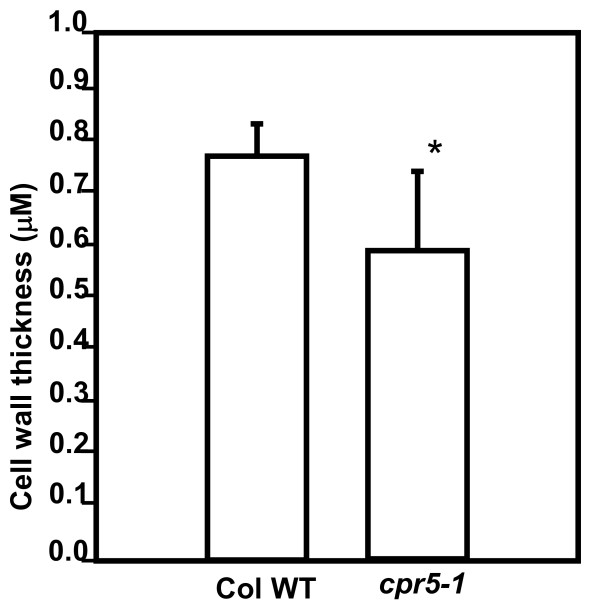
**Walls between adjoining leaf adaxial epidermal cells are thinner in *cpr5-1 *plants than in wild-type.** N = 20 cells; error bars show standard deviation. * indicates a significant difference in a paired T-test, p < 0.05.

Several other mutants with transparent "glassy" trichomes have been described [5,12]. For the best characterized of these, *trichome birefringence *(*tbr*), reduced birefringence of trichome cell walls was associated with reduced paracrystalline cellulose in leaves [12]. As determined by the chemical method of Updegraff [13], there was significantly less paracrystalline cellulose in the walls of *cpr5-1 *rosette leaves (p < 0.002), with walls of the mutant containing approximately 38% of the paracrystalline cellulose of wild-type walls (Figure 3A). The cell wall monosaccharide composition of rosette leaf cell walls was also determined. No qualitative differences were found, though small increases in xylose (p < 0.05) and arabinose (p < 0.01) were observed in *cpr5-1 *relative to wild-type (Fig. 3B). The thickness of cell walls between adjoining epidermal cells of *cpr5-1 *and wild-type was directly examined by TEM. The *cpr5-1 *mutant was found to have slightly but significantly thinner walls than wild-type (p < 0.01, Fig. 4).

### Genetic interactions between *cpr5 *and genes involved in trichome initiation

To gain further insight into the role of *CPR5 *in trichome development, *cpr5-1 *and *cpr5-2 *mutants were crossed with mutant plants for the trichome developmental regulator *try*, which has effects on trichome size, branching, and endoreplication opposite those of *cpr5 *mutants. The *TRY *gene encodes a Myb protein that acts as an inhibitor of trichome development. The mutation in the *try-JC *allele used here results in a protein truncated in the middle of the conserved Myb DNA-domain [14], and is phenotypically a strong loss-of-function allele.

**Figure 5:**
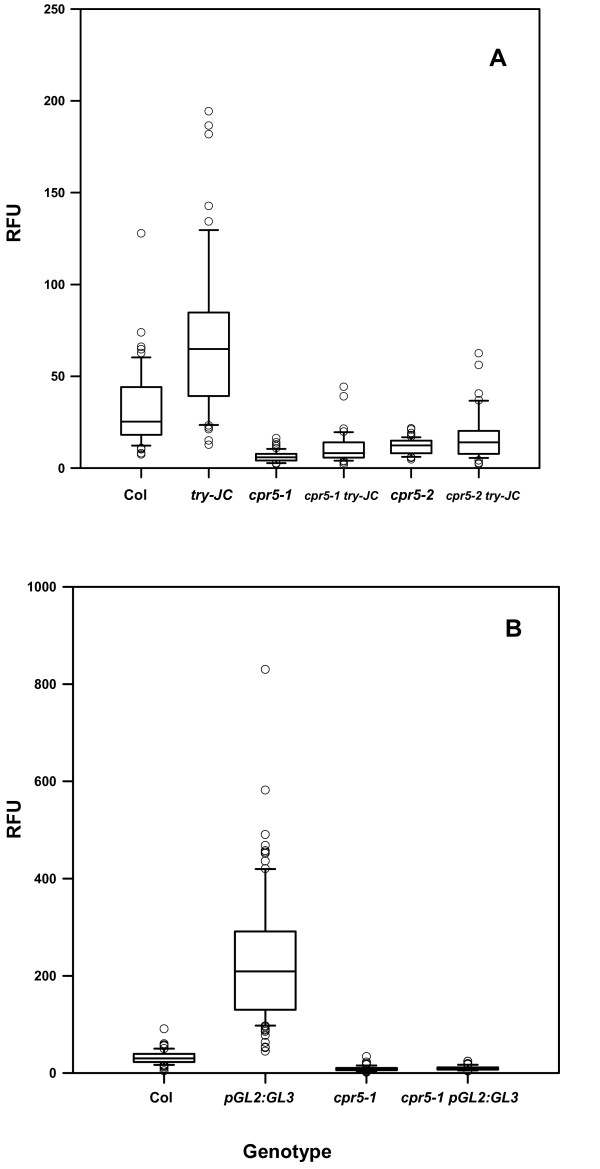
**In situ measurements of trichome DNA contents of *cpr5 *single and double mutants.** (A) Interaction of *cpr5 *alleles and *try*. (B) Interaction of *cpr5-1 *and *proGL2:GL3*. DNA contents of DAPI-stained nuclei are presented as Relative Fluorescent Units (RFU), normalized to 32 RFU for Col-0, based on an assumed DNA content of 32C for wild-type trichome nuclei. Measured RFU values should thus correspond approximately to DNA contents. Data are presented as Box Plots, where the box encompasses the 25th through the 75th percentile of the data, the line within the box is the median (50th percentile), and the error bars represent the 5th (lower bar) and 95th (upper bar) percentiles. Statistical analysis is given in Tables 2 and 4. For the *proGL2:GL3 *genotype in (B), a single data point at RFU = 1600 was omitted from the Figure for clarity of presentation, though this point was included in the analysis in Table 4.

Trichomes of *try-JC *plants are larger and more branched than those of wild-type plants, and highly birefringent (Fig. 1A, D). In contrast, the trichomes of double mutant *cpr5-1 try-JC *and *cpr5-2 try-JC *plants are similar in size to those of corresponding *cpr5 *allele (Fig. 1B, C, E, F), indicating that *cpr5 *is epistatic to *try *with regard to trichome size. The reduced DNA content of *cpr5 *trichome nuclei is also clearly epistatic to the increased DNA content of *try-JC *trichome nuclei (Fig. 5A, Table 2).

**Table 2:** DNA contents of trichome nuclei for interactions of *cpr5 *with *try *and *proGL2:GL3*.

Genotype	Median DNA content (RFU)	Mean DNA content ± s.d.	N
Col	25.3	32 ± 20.8	57
*try-JC*	64.9	71.1 ± 42.4	57
*cpr5-1*	5.8	6.2 ± 3.1	53
*cpr5-1 try-JC*	8.1	11.0 ± 8.6	41
*cpr5-2*	12.3	11.8 ± 4.2	56
*cpr5-2 try-JC*	14.0	16.7 ± 12.6	48

RFU = relative fluorescence units. s.d. = standard deviation. N = number of nuclei examined. RFU values have been normalized to 32, the expected value for trichome nuclei of the wild-type Col strain, and thus RFU values should roughly correspond to DNA contents. A Kruskal-Wallis One Way ANOVA indicated that differences in the medians were greater than expected by chance (p < 0.001); an all pairwise multiple comparison (Dunn's Method) indicated that all pairwise comparisons were significantly different (p < 0.05) except *cpr5-1 *vs. *cpr5-1 try-JC *and *cpr5-2 *vs. *cpr5-2 try-JC*.

Unlike the trichomes of wild-type plants, *try *trichomes often occurred in clusters of immediately adjacent trichomes due to failure of lateral inhibition signaling (Fig. 1D). Like wild-type trichomes, trichomes on *cpr5 *mutant leaves only rarely occurred in clusters (Fig. 1A, B, C, Table 3). Trichomes of the *cpr5 try *double mutants frequently occur in clusters (Fig. 1E, F), indicating that *cpr5 *was not epistatic to this aspect of the *try *phenotype. However, closer inspection revealed an unexpected synthetic genetic interaction whereby the *cpr5 *mutations increased the number of trichomes in each trichome cluster above that seen for *try-JC *alone (Table 3). The percentage of trichomes in clusters on *cpr5-1 try-JC *and *cpr5-2 try-JC *leaves was approximately double the percentage on *try-JC *leaves (Table 3). This difference was due primarily to an increase in the number of trichomes in each cluster for each of the *cpr5 try *double mutants relative to *try-JC *(p < 0.001 for the comparison of either double mutant with *try-JC*), which averaged nearly three trichomes per cluster, compared to two trichomes per cluster in the *try-JC *single mutant (Table 3, p < 0.001 for the comparison of either double mutant with *try-JC*, and Fig. 1D,E,F).

**Table 3:** Frequencies of trichome initiation sites and trichome clusters in *cpr5*, *try *and *cpr5 try *double mutants.

Genotype	Mean # of trichomes/leaf	Mean # of TIS/leaf	Mean % of trichomes in clusters	Mean # of trichomes/cluster
Col	47.2 ± 6.6	47.1 ± 6.7	0.4	2
*try-JC*	36.1 ± 6.8	31.8 ± 6.1	23.5	2.1 ± 0.4
*cpr5-1*	44.2 ± 4.0	44.2 ± 4.0	0	0
*cpr5-1 try-JC*	58.4 ± 8.3	36.8 ± 3.4	56.2	2.9 ± 0.4
*cpr5-2*	44.6 ± 5.3	44.5 ± 5.3	0.3	2
*cpr5-2 try-JC*	61.7 ± 8.5	39.1 ± 6.8	58.7	2.7 ± 0.2

TIS = Trichome initiation site; a site on the leaf where one or more contiguous trichomes originate. All trichomes were counted on 10–15 first leaves per genotype.

**Figure 6:**
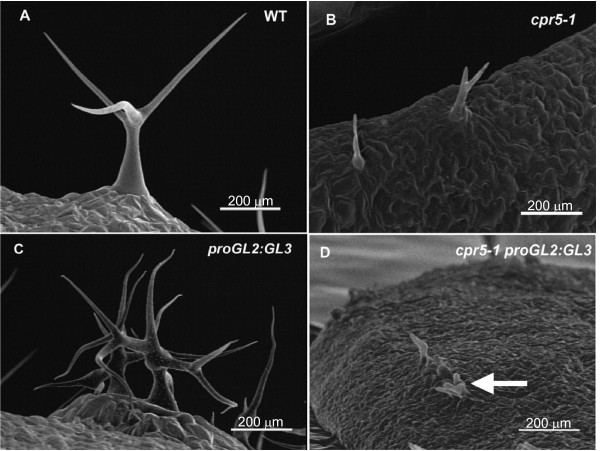
**Trichome phenotypes of *proGL2:GL3 *and *cpr5-1 proGL2:GL3*.** Images are scanning electron micrographs; all scale bars are 200 μm. (A) Col-0 wild-type, (B) *cpr5-1*, (C) *proGL2:GL3*, (D) *cpr5-1 proGL2:GL3*.

Plants expressing the trichome developmental regulator *GL3 *from the strong and relatively trichome-specific *GL2 *promoter (*proGL2:GL3*) had large, highly birefringent trichomes with increased branching (Fig. 6C), similar to *try-JC *trichomes. Plants of the genotype *cpr5-1 proGL2:GL3 *have small trichomes similar to those of *cpr5-1 *plants (Fig. 6B,D), indicating that *cpr5 *is also epistatic to the increased trichome size conditioned by this construct. Wild-type plants containing the *proGL2:GL3 *construct also endoreplicate trichome nuclear DNA to very high levels, on the order of ten times that of wild-type (Fig. 5B, Table 3). The *cpr5-1 proGL2:GL3 *double mutant has a DNA content similar to that of the *cpr5-1 *single mutant, indicating that *cpr5 *is epistatic to *proGL2:GL3 *with regard to the degree of endoreplication (Fig. 5B, Table 4).

**Table 4:** DNA contents of trichome nuclei in *cpr5-1*, *proGL2:GL3 *and *cpr5-1 proGL2:GL3*.

Genotype	Median DNA content (RFU)	Mean DNA content ± s.d.	N
Col	27.0	32 ± 17.8	60
*proGL2:GL3*	321.7	370.4 ± 225.9	60
*cpr5-1*	8.2	9.1 ± 4.8	60
*cpr5-1 proGL2:GL3*	12.0	11.9 ± 5.8	60

RFU = relative fluorescence units. s.d. = standard deviation. N = number of nuclei examined. RFU values have been normalized to 32, the expected value for trichome nuclei of the wild-type Col strain, and thus RFU values should roughly correspond to DNA contents. A Kruskal-Wallis One Way ANOVA indicated that differences in the medians were greater than expected by chance (p < 0.001); an all pairwise multiple comparison (Tukey Test) indicated that all pairwise comparisons were significantly different (p < 0.05) except *cpr5-1 *vs. *cpr5-1 proGL2:GL3*.

## Discussion and Conclusion

*CPR5 *has been variously proposed to play specific roles in pathogen response signaling [1], senescence [4], and cell proliferation and cell death [3]. The work presented here identifies a previously unrecognized cell wall defect in *cpr5 *mutants, resulting in a deficit of paracrystalline cellulose. At the same time, the epistasis of the *cpr5 *phenotype in genetic interactions with *try *and *proGL2:GL3 *indicates that *CPR5 *function is necessary for the increased cell expansion and endoreplication conditioned by loss of *TRY *function or by over-expression of *GL3*. These two genes encode transcription factors that play opposing roles in trichome development. Unexpectedly, *cpr5 *mutations also appear to enhance the lateral inhibition signaling defect of *try *mutants that normally prevents trichomes from forming adjacent to one another (Table 2).

It is difficult to reconcile our results with the specific roles that have been proposed by others as the most fundamental function of the *CPR5 *gene product, such as pathogen response signaling or endoreplication and programmed cell death. No other constitutive pathogen response mutants have been reported to affect trichomes, and we have observed no trichome defects on examining *constitutive expressor of pathogenesis-related genes1*, *nonexpressor of PR genes1 *(*npr1*), *accelerated cell death2 *(*acd2*), and *accelerated cell death6 *(*acd6*), and these mutants appeared to have normal birefringent trichome cell walls (J. C. Larkin, unpublished observations). Similarly, *gl3 *loss-of-function mutants, which have reduced endoreplication, produce normally birefringent trichome walls [5], and trichome walls of plants expressing *proGL2:ICK1/KRP1*, a construct that induces programmed cell death in trichomes [15], also appear to be normal (R. Kasili and J. C. Larkin, unpublished observations). It thus seems likely that the *CPR5 *gene product is involved in some general process that is indirectly necessary for trichome cell expansion, pathogen response signaling, and suppressing premature senescence and programmed cell death, rather than being a specialized component of any one of these processes.

An attractive locus of action for the *CPR5 *gene product suggested by our data is the cell wall itself. The cell wall is directly involved in several processes related to the *cpr5 *mutant phenotype, including both cell expansion and pathogen responses [16]. Recently, a reduction of the rhamnogalacturonan II component of tobacco cell walls by Virus-induced Gene Silencing (VIGS) of a UDP-D-apiose/UDP-D-xylose synthase gene was shown to result in dwarfing of plants, induction of several pathogen-response genes, production of reactive oxygen species, and cell death [17]. A *cev1 *mutant of Arabidopsis, which has a mutation in the *CES3A *cellulose synthase gene, was also shown to result in constitutive expression of pathogen response genes and to have enhanced pathogen resistance [18]. This mutant was originally identified as an activator of jasmonic acid signaling pathways, and overproduces jasmonic acid and ethylene. Furthermore, a mutation in another cellulose synthase gene, *rsw1*, also results in activation of jasmonic acid signaling, and cellulose biosynthesis inhibitors can mimic the *cev1 *mutant phenotype in wild-type plants, including the activation of pathogen response genes [18]. Jasmonic acid-dependant pathogen response pathways are known to be activated in *cpr5 *mutants [1,19]. And, not surprisingly, mutations in cellulose synthase genes can affect cell morphology and expansion [20,21]. Indeed, the cell expansion defects seen in *cpr5 *mutants are comparable in severity to those seen in cellulose synthase mutants [3,22]

Taken together, these results demonstrate that defects in the cell wall itself can lead to many of the specific aspects of the *cpr5 *mutant phenotype, including the constitutive pathogen response signaling for which it is named. One aspect of the phenotype that is less obviously coupled to the cell wall is the reduced endoreplication seen in *cpr5 *[3]. However, the degree of endoreplication is often strongly correlated with cell size [6], and it is possible that limitations on cell expansion have a feedback effect on endoreplication.

An alternative model to explain the extreme pleiotropy of the *cpr5 *mutant phenotype that is the CPR5 protein may be required directly for the function of multiple transcription factors involved in a wide range of distinct processes. Our observation that *cpr5 *is epistatic to the phenotypes conditioned by *try *loss-of-function or *GL3 *overexpression indicates that *CPR5 *function is required for the cell expansion and increased endoreplication conditioned by these two transcription factors. The *CPR5 *gene product is predicted to be a Type IIIa membrane protein with five transmembrane domains and a cytoplasmic N-terminus. This N-terminal domain contains a bipartite nuclear localization sequence (NLS), and it has been proposed that the protein may be involved in a signaling cascade in which the cytoplasmic domain is proteolytically cleaved and transported into the nucleus [3], a signaling process for which there is substantial precedent [23,24]. An alternative is suggested by recent work demonstrating that some membrane proteins in yeast that localize to the inner nuclear membrane are targeted to this membrane via an NLS and use an karyopherin-dependant pathway to enter the nucleus [25]. In this case, the full-length CPR5 protein might be directly required in the nucleus for function of multiple transcription factors.

Either of these models, a primary role for CPR5 in cell wall biogenesis or a primary role for CPR*5 *in regulation of nuclear transcription, can provide an explanation for the reduced lateral inhibition (i.e., increased trichome cluster size) seen in the *cpr5 try *double mutants (Table 3). Altered cell wall structure could reduce transmission of the inhibitory signal by reducing intercellular transport of the functionally redundant members of the TRY protein family, CPC, ETC1, and ETC2, perhaps by altering plasmodesmata. Alternatively, if CPR5 is needed for function of the GL3-TTG-GL1 transcriptional activation complex in the nucleus, reduced activity of this complex might result in inefficient upregulation of these TRY homologs in developing trichomes, reducing the degree of inhibition of trichome development in neighboring cells.

It is obvious that testing these models will require biochemical analysis of localization and function of the CPR5 protein. Perhaps because it is a membrane protein, little progress has been reported on this front, and our own attempts in this regard have not been fruitful. For example, fluorescent protein fusions to the *CPR5 *coding sequence were generated that fully complemented the *cpr5 *mutant phenotype, but no fluorescence was detected in any of the transgenic lines (V. Kirik, unpublished observations). Nevertheless, the work presented here suggests that the cell wall may be a unifying locus for CPR5 function, and will provide guidance for further studies. The important role of *CPR5 *in multiple essential aspects of plant growth and development merits further work to unravel the mechanism of *CPR5 *function.

## Methods

### Plant materials and growth conditions

Plants were grown under constant illumination as described previously. All alleles originated in the Columbia ecotype, which was used as the wild-type for these studies, and all alleles had been backcrossed to Columbia at least twice prior to use in this work. The *cpr5-1 *allele was obtained from Dr. Xinnian Dong [1]; *cpr5-2 *derives from our previous work [2]. The *try-JC *allele has been described previously [14,26]; in the work of Schellman et al. [14], it is mis-labeled as the "*try-5C*" allele. The identity of the *cpr5 try-JC *double mutants was confirmed by the failure of the double mutants to complement either parent mutant after crossing. The early senescence of *cpr5 *cotyledons was maintained in the double mutants and aided in identifying them. The *proGL2:GL3 *construct has been previously described [27].

### Carbohydrate analysis

For carbohydrate analysis, uncrowded plants just prior to bolting were placed in the dark for 24–48 hours to reduce the amount of starch in the leaves. The paracrystaline cellulose content of ethanol-washed cell walls (three washes of 70% ethanol at 70°C) of rosette leaves was determined by the method of Updegraff [13], using cellulose from Sigma as the standard.

For analysis of non-cellulosic wall monosaccharides, cell wall material was prepared by grinding leaf tissue in 80% ethanol, washing residue with 80% ethanol, then 100% ethanol, treating residue for 30 minutes with methanol:chloroform (1:1 v/v), washing residue with acetone, and air drying the residue. Further preparation and monosaccharide composition analysis was provided by the Complex Carbohydrate Research Center at the University of Georgia, Athens, GA. This included hydrolysis using freshly prepared 1 M methanolic-HCl for 16 hours at 80°C and derivatization of the released sugars with Tri-Sil. The samples were analyzed by GC using a Supelco column. Myo-inositol was added as an internal standard.

### Electron Microscopy

Samples fixed in FAA (3.7% formaldehyde, 50% ethanol, 5% acetic acid) were prepared for scanning electron microscopy by standard methods, as described previously [26]. For TEM, leaves were fixed in 2% glutaraldehude and 1% paraformaldehyde in 0.2 M cacodylate buffer (pH 7.4) at room temperature for 2 hours, then rinsed with 0.1 M cacodylate buffer and postfixed in buffered 1% osmium tetroxide (OsO_4_) for 1 hour. After staining with 1% uranyl acetate for 1 hour, the materials were dehydrated in an ethanol series and embedded in LR White resin (medium grade). Thin sections (70–90 nm) were stained with lead citrate, and observed and photographed with a JEOL 100 X transmission electron microscope.

### Light Microscopy

Nuclear DNA contents were measured and normalized to a level of 32C for wild-type trichome nuclei essentially as described previously [28,29], except that samples were observed with the 200 × objective of a Leica DM RXA2 microscope, and images were captured with a SensiCam QE 12-bit, cooled CCD camera and analyzed with Slidebook software from 3I. Care was taken when setting image capture parameters that the nuclei with the highest DNA content in a group of samples did not saturate the dynamic range of the images. Non-parametric statistics (Kruskal-Wallis One Way ANOVA and Dunn's all pairwise multiple comparison) were performed using SigmaStat. Birefringence was examined using a Nikon FXA microscope equipped with a SpotCam. Samples were cleared with 95% ethanol and placed on a circular rotating stage between two polarizing filters, the polarizer and the analyzer, that were oriented at right angles to each other.

## Authors' contributions

GB backcrossed the *cpr5 *alleles, generated the *cpr5 try *double mutants, carried out the cell wall biochemistry, did much of the electron microscopy, prepared several figures, and drafted the manuscript. RK did the scanning electron microscopy of the *pGL2:GL3*-containing lines and contributed to the DNA content determinations. LAS did the DNA content determinations and statistical analysis, and prepared the final versions of the figures. VK constructed the *pGL2:GL3 cpr5-1 *line and did the initial analysis on this line, and contributed to drafting the manuscript. MH was involved in the design of initial studies with the *pGL2:GL3 cpr5-1 *line and helped with the manuscript. JCL participated in the design of the study, did the work on birefringence of *cpr5 *trichomes, and helped draft the manuscript. All authors have read and approved the final manuscript.
